# Independent prognostic factors for posttransplant survival in hepatocellular carcinoma patients undergoing liver transplantation

**DOI:** 10.1002/cam4.936

**Published:** 2016-11-16

**Authors:** Minzhi Xing, Hyun S. Kim

**Affiliations:** ^1^ Interventional RadiologyDepartment of Radiology and Biomedical ImagingYale School of MedicineNew HavenConnecticut; ^2^ Yale Cancer CenterNew HavenConnecticut

**Keywords:** Liver Transplantation, Hepatocellular Carcinoma, Bridging Locoregional Therapy, Survival

## Abstract

The aim of this study was to investigate longitudinal trends in locoregional therapy (LRT) use in hepatocellular carcinoma (HCC) patients listed for transplant, and evaluate independent prognostic factors for overall survival (OS) in HCC patients undergoing orthotopic liver transplantation (OLT). The United Network for Organ Sharing (UNOS) database was used to identify HCC patients listed for liver transplantation from 1988 to 2014, and longitudinal rates of bridging LRT were calculated. OLT recipients listed from 2002 to 2013 and transplanted up to 2014, with ≥1 year of follow‐up were further analyzed. OS was compared between patients receiving bridging LRT versus none, high versus low wait times (HWT vs. LWT), and by geographic region. Bridging LRT use in the US has increased dramatically over 25 years, with more than 50% of listed patients receiving at least 1 LRT in 2014. Of 17,291 HCC patients listed for LT from 2002 to 2013, 14,511 received OLT, mean age 57.4 years, 76.8% male; 3889 received bridging LRT. Comparison groups were similar for gender, race, body mass index (BMI), HCC etiology, and biological MELD scores (*P* > 0.05). Significant differences in mean OS in regions with HWT/high LRT (122.4 months), HWT/low LRT (104.5 months), LWT/high LRT (104.2 months), and LWT/low LRT (102.3 months) were observed, *P* = 0.0006. Recipient age, donor age, bridging LRT, and longer wait times were independent prognostic factors of survival from OLT. Increasing longitudinal trends in bridging LRT for HCC patients were observed. Younger age, younger donor age, high wait times, and bridging LRT were significant independent prognostic factors for prolonged survival from transplant.

## Introduction

Since the widespread implementation of the Milan criteria in 1996, liver transplantation (LT) has been acknowledged as the best curative option for patients with hepatocellular carcinoma (HCC) 1. From 2002, exception points were granted for HCC orthotopic liver transplant (OLT) recipients based on risk of tumor progression and thus waitlist dropout with the adoption of the Model for End‐stage Liver Disease (MELD) scoring system. Subsequently, a significant increase in the proportion of such patients receiving OLT for HCC was observed 2.

In order to increase equity among candidates, numerous improvements in MELD allocation exception point system have been suggested and implemented 3. However, despite such improvements, current data suggest that HCC recipients are over‐prioritized in the allocation system, with lower rates of waitlist dropout and higher transplant rates than non‐HCC patients despite inferior posttransplant survival 4, 5. Up until September 2015, all eligible HCC recipients meeting specified criteria were immediately granted 22 MELD exception points, with 10% increases at 3‐month intervals. However, multiple studies have indicated that patients have variable individual rates of tumor progression, waitlist dropout 6, 7, 8, and posttransplant recurrence based on patients' individual tumor biology 9.

Based on such evidence, the revised Organ Procurement and Transplantation Network (OPTN) policy implemented in October 2015 capped the maximum HCC exception score at 34 and established a new timetable for a 6‐month delay before assignment of an exception MELD score of 28, effectively extending waitlist time for potential HCC recipients 10. Although prolonging HCC waitlist times by 6 to 9 months has been demonstrated to improve equity and access in organ allocation modeling 6, its effect on post‐LT survival is not well characterized.

Pretransplant locoregional therapies (LRTs) such as transarterial treatments (transarterial chemoembolization, TACE; and radioembolization, TARE) and percutaneous thermal ablation (radiofrequency ablation, RFA; microwave ablation) have been used widely in transplant centers to bridge and/or downstage HCC recipients prior to LT. LRT aims to achieve pathologic tumor necrosis, with reported rates of complete pathologic response in 27% to 57% of patients after TACE 11, 12 and 47% to 75% after thermal ablation 13, 14, 15. While data for LRT reducing risk of tumor progression and waitlist dropout has been widely published 13, 14, 15, 16, there is limited and contradictory evidence with regards to LRT effectiveness for reducing posttransplant HCC recurrence and improving posttransplant survival 17, 18. The aim of this study was to investigate independent prognostic factors for posttransplant survival in HCC LT recipients using a national transplant database.

## Materials and Methods

This was an IRB‐approved, Health Insurance Portability and Accountability Act (HIPAA) compliant study. The Standard Transplant Analysis and Research (STAR) registry of the United Network for Organ Sharing (UNOS) current through October 2015 was utilized. Data were collected on 239,127 patients who were listed for liver transplantation (LT) between 1988 and 2014. Patients within the Milan criteria for whom an HCC Model for End‐Stage Liver Disease (MELD) exception was approved were included. Liver recipients aged <18 years at time of LT, those who dropped out from the waiting list, patients with OPTN status 1 indications, living donor liver transplantation (LDLT) recipients, split or partial liver recipients, multi‐organ recipients, prior LT recipients, and patients receiving MELD prioritization for non‐HCC indications were excluded.

**Figure 1:**
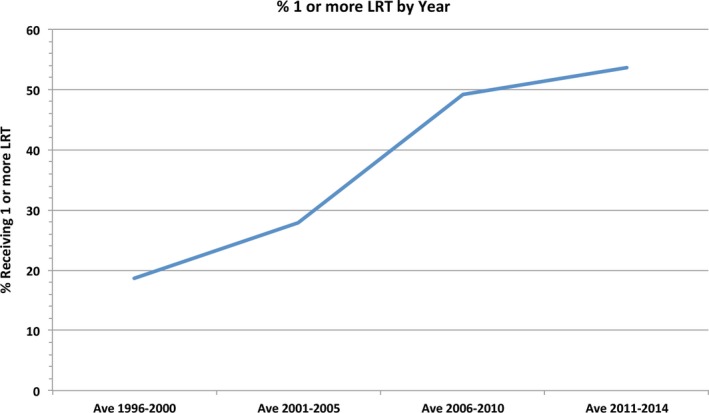
Proportion of hepatocellular carcinoma patients listed for liver transplantation who received 1 or more sessions of locoregional therapy by year of listing.

**Figure 2:**
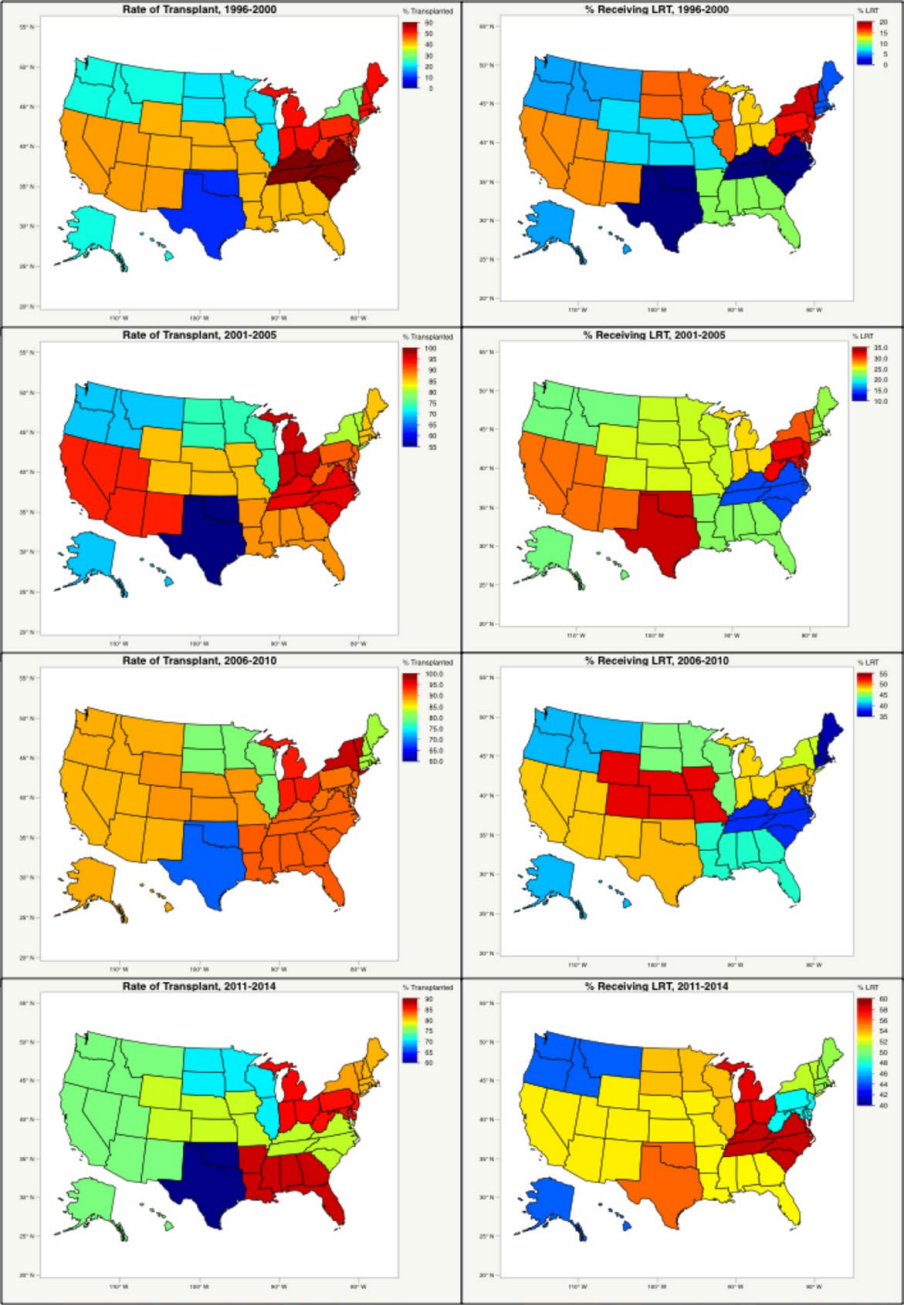
Proportion of patients treated with locoregional therapy and rate of transplant by United Network for Organ Sharing region.

The proportion (%) of patients who received bridging LRT were stratified based on treatment year from 1996 to 2014 (Fig. 1). Using the most recent UNOS data from 2011 to 2014, the mean rate of transplantation and mean rate of LRT were separately compared between each UNOS region (Table 1). Transplant rates in regions with high LRT use verses low LRT use were compared via chi‐squared test. The rate of LRT use (at least one therapy) and rate of transplant was stratified by UNOS region and year of treatment from 1996 to 2014 (Fig. 2).

**Table 1:** Mean transplant and locoregional therapy (LRT) rates based on United Network for Organ Sharing region

Region	Mean rate of transplant, 2011–2014, %	*P*‐value	Mean LRT rate, 2011–2014, %	*P*‐value
Overall	78.06	**–**	52.60	**–**
1	80.92	**<0.0001**	51.08	**<0.0001**
2	85.90		47.57	
3	87.72		52.59	
4	60.20		55.53	
5	74.97		52.79	
6	74.87		44.36	
7	70.87		53.69	
8	77.86		52.76	
9	81.47		51.77	
10	86.21		57.94	
11	77.65		58.54	

Bold text indicates statistically significant *P*‐values

Outcomes were analyzed in a study population comprising adult patients with HCC who fulfilled the MELD exception criteria for transplant eligibility, registered on the LT waitlist between 2002 and 2013, received a first liver transplantation between January 2002 and January 2014, and had at least one year of post‐LT follow‐up. Liver recipients aged <18 years at time of LT, those who dropped out from the waiting list, patients with OPTN status 1 indications, living donor liver transplantation (LDLT) recipients, split or partial liver recipients, multi‐organ recipients, prior LT recipients, and patients receiving MELD prioritization for non‐HCC indications were excluded.

Retrieved characteristics of the population were age at OLT, donor age, gender, ethnicity, body mass index (BMI), donor BMI, etiology of HCC, time spent on the waiting list, transplant region and center, warm ischemic time of graft, tumor size, locoregional therapy status and functional status at transplant. Bridging locoregional therapy included recipients who received at least one of any of the following prior to LT: transarterial chemoembolization (TACE), radiofrequency ablation (RFA), thermal ablation (TA), cryoablation (CRA), or radiation microspheres.

Comparison was made between overall post‐LT survival of recipients who underwent bridging locoregional therapy versus recipients who underwent none; and between recipients with high waitlist time (HWT; defined as >180 days) versus low waitlist time (LWT; ≤180 days). Geographical differences between states based on waitlist times and bridging locoregional therapy use were identified. Characteristics for each UNOS allocation region, including mean waitlist times and bridging locoregional therapy rates (high LRT defined as regions with greater than overall mean % receiving LRT) were analyzed. Overall post‐LT survival of recipients stratified by UNOS regions based on both waitlist time and rate of LRT use was evaluated. Independent prognostic factors for increased post‐LT survival were identified.

### Statistical analysis

Categorical baseline variables were compared via chi‐squared test; continuous variables were compared via the Student *t*‐test if normally distributed, and with the rank‐sum test if not normally distributed. Using the Kaplan–Meier method, mean overall survival, 1‐, 3‐, and 5‐year actuarial survival rates, and associated 95% confidence intervals (CIs) were calculated. Mortality rates were compared with relative risks (RRs), and estimated survival functions were compared with the log‐rank test. Post‐LT survival analysis was performed via Cox proportional hazards regressions for all potential predictors of survival, with variables selected for inclusion in the multiple regression model using stepwise methods (*P*‐value thresholds of 0.05 for inclusion and 0.10 for elimination). A two‐sided *P*‐value of <0.05 was held to be statistically significant. All calculations were performed using SAS v9.4 (SAS Institute, Cary, NC).

## Results

### Longitudinal trends in bridging locoregional therapy in waitlisted patients

The proportion of patients undergoing 1 or more bridging LRT procedures increased over time from an average of 18.6% (1996–2000) to 53.7% (2011–2014) (Fig. 1). Overall, TACE was the most common LRT modality used in 62.7% of patients who received bridging therapy, whereas RFA was used in 16.9% of patients. A significant difference in the mean rate of bridging LRT use between all regions (*P* < 0.001) (Table 1) was observed in the most recent UNOS data from 2011 to 2014. LRT rates ranged from LRT rates ranging from 44.36% in Region 6 to 58.54% in Region 11 (*P* < 0.0001). Similarly, significant differences in mean rates of transplant were observed between UNOS regions in the same time period (*P* < 0.001) (Table 1). Transplant rates ranged from 70.87% in Region 7 to 86.21% in Region 10 (*P* < 0.0001). A comparison of transplant rate between regions with low LRT rates (mean 49.47%) versus high LRT rates (mean 55.21%, *P* = 0.06).

### Outcomes analysis in transplanted patients

**Figure 3:**
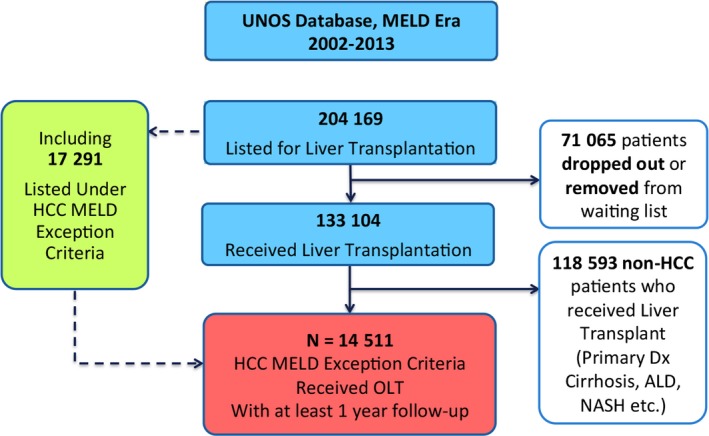
Study flowchart.

Of 17,291 candidates listed under the HCC MELD exception criteria for liver transplantation between 2002 and 2013, 14,511 HCC recipients met the inclusion criteria for this study (Fig. 3), including 2926 who received TACE, and 1118 who received RFA.

Overall baseline study characteristics of the study population and comparisons between those who received bridging LRT versus no LRT, and who had high versus low waitlist times are summarized in Table 2. No significant differences between those who received bridging LRT versus no LRT in terms of age of recipient, donor age, gender, donor gender, ethnicity, BMI, donor BMI, etiology of HCC, mean MELD score, number of tumors, mean largest tumor size, median wait time, warm ischemic time of graft, and functional status at LT were identified (*P* > 0.05 for all parameters). Median time on the waitlist was 384.5 days for recipients in the HWT group, as compared to 56.0 days in the LWT group, *P* < 0.001. No other factors were found to be significantly different between HWT and LWT groups at baseline (*P* > 0.05 for all parameters).

**Table 2:** Baseline patient characteristics

*N* = 14,511		Bridging locoregional therapy (LRT)	No bridging LRT	*P*‐value	High wait time	Low wait time	*P*‐value
Age at orthotopic liver transplantation	Mean (SD)	57.80 (7.15)	57.23 (8.05)	0.54	57.22 (7.94)	57.62 (7.64)	0.66
Donor age	Mean (SD)	42.07 (16.85)	41.93 (16.93)	0.51	42.33 (16.83)	41.97 (17.62)	0.53
Gender	Male	80.1%	75.6%	0.67	74.7%	78.9%	0.77
	Female	19.9%	24.4%		25.3%	21.1%	
Donor gender	Male	61.5%	58.9%	0.09	59.5%	59.7%	0.83
	Female	38.5%	41.1%		40.5%	40.3%	
Ethnicity	White	69.4%	66.4%	0.31	64.2%	70.1%	0.15
	Black	10.0%	8.7%		8.1%	10.0%	
	Other	20.7%	24.9%		27.8%	20.0%	
BMI	>25	75.5%	73.9%	0.24	74.7%	74.0%	0.25
	≤25	24.5%	26.1%		25.3%	26.0%	
Donor BMI	>25	59.8%	59.2%	0.31	59.3%	59.4%	0.84
	≤25	40.2%	40.8%		40.7%	40.6%	
Etiology of hepatocellular carcinoma (HCC)	Hepatitis B	30.7%	31.3%	0.25	33.1%	29.3%	0.08
	Hepatitis C	40.5%	38.1%		37.3%	40.1%	
	Other	28.8%	30.6%		29.6%	30.6%	
Mean MELD score (SD)		29.6	28.7	0.23	30.8	29.1	0.11
Number of lesions	≤3	55.3%	57.2%	0.55	62.6%	60.3%	0.42
	>3	44.7%	42.8%	0.48	37.4%	39.7%	0.39
Mean largest tumor size, cm ± SD		3.02	2.16	0.06	2.57	2.63	0.38
Serum AFP (ng/mL)	<400	94.2%	92.4%	0.14	95.5%	92.6%	0.13
	≥400	5.8%	7.6%		4.5%	7.4%	
Milan criteria	Within Milan	63.8%	71.7%	0.08	72.9%	70.3%	0.11
	Beyond Milan	36.2%	28.3%		27.1%	29.7%	
Median wait time (days)		101.3	185.0	0.15	384.5	56.0	**<0.001**
Warm ischemic time	>30 min	81.0%	82.7%	0.26	83.7%	81.3%	0.06
	≤30 min	19.0%	17.3%		16.3%	18.7%	

Bold text indicates statistically significant *P*‐values

**Figure 4:**
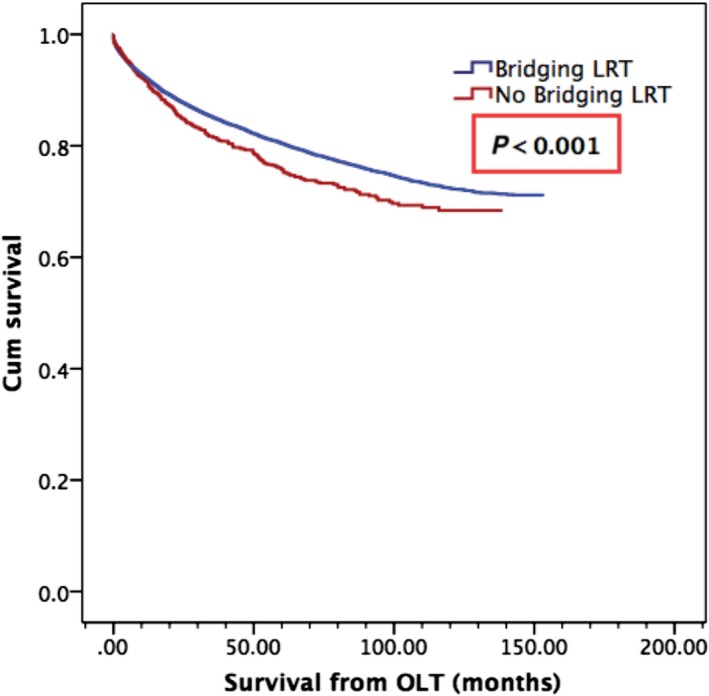
Overall survival from orthotopic liver transplantation by bridging locoregional therapy (LRT) status. Mean survival of 122.1 months in those who received pretransplant bridging LRT, as compared to 108.7 months in those who did not, *P* < 0.001.

**Figure 5:**
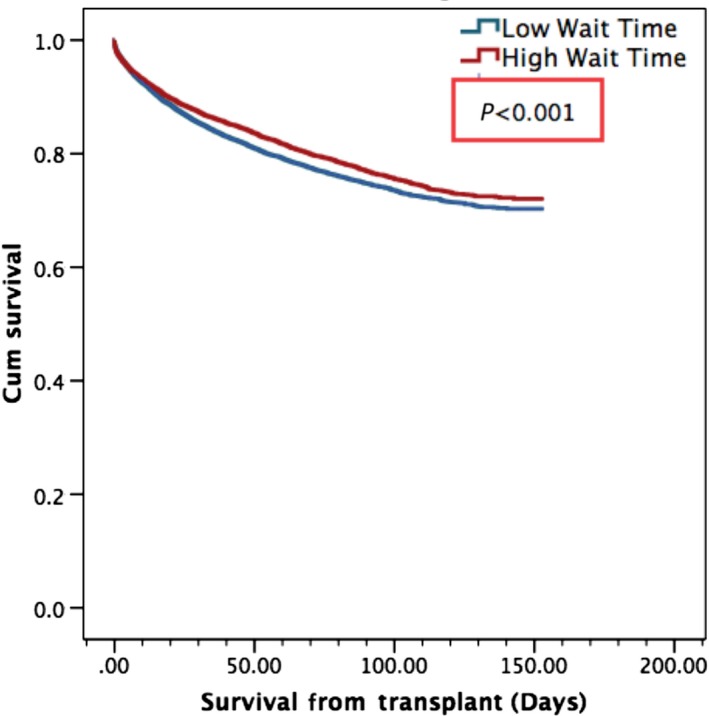
Overall Survival from orthotopic liver transplantation by wait time. Mean survival of 123.3 months in patients who had wait times in excess of 180 days or 6 months, as opposed to 120.4 months in those with low wait time, *P* < 0.001.

Hepatocellular carcinoma transplant recipients who received bridging LRT were found to have significantly greater mean overall survival from LT (122.1 months) compared to those who received no LRT (108.7 months), *P* < 0.001. Actuarial survival rates for LRT versus no LRT groups were as follows: 95% versus 94% (1‐year), 85% versus 83% (3‐year), 80% versus 78% (5‐year), *P* < 0.001 (Fig. 4). Recipients who had high waitlist times (>180 days) had a significantly higher mean overall survival from LT (123.3 months) versus those who had low waitlist times (120.4 months), *P* < 0.001. Actuarial survival rates for HWT versus LWT groups were as follows: 94% versus 93% (1‐year), 86% versus 81% (3‐year), 80% versus 76% (5‐year), *P* < 0.001 (Fig. 5).

**Figure 6:**
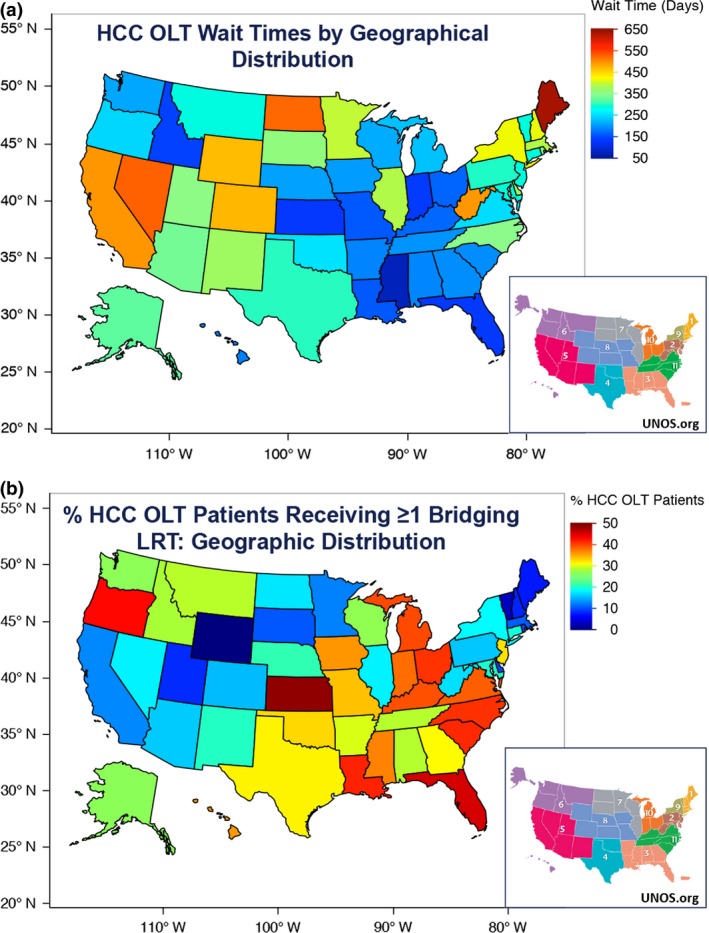
(A) Geographical distribution of study population based on mean waitlist times. (B) Geographical distribution of the rate of Bridging locoregional therapy use.

**Figure 7:**
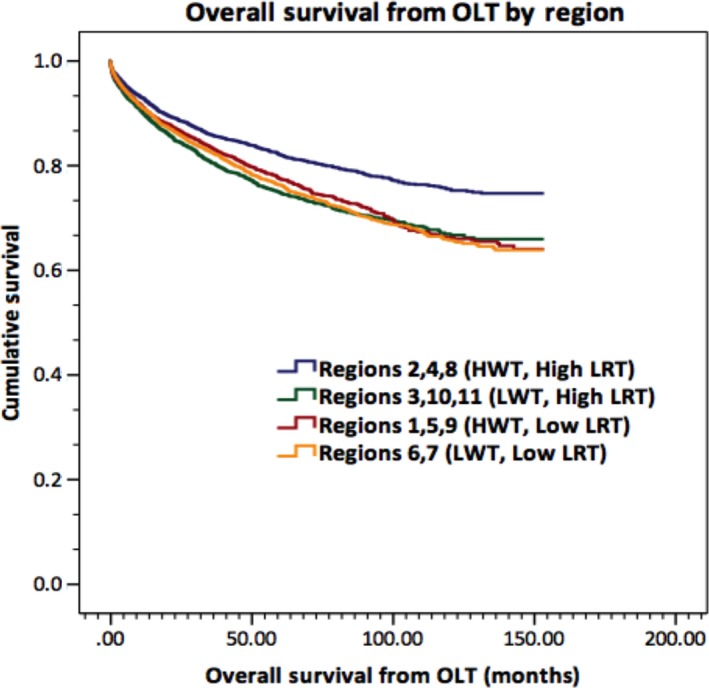
Overall survival from orthotopic liver transplantation by region locoregional therapy (LRT) status and waitlist time. Mean survival was 124.9 months in high waitlist time (HWT), High LRT regions; 114.6 months in low wait times (LWT), High LRT regions; 116.0 in HWT, Low LRT regions; and 114.8 months in LWT, Low LRT regions, *P* = 0.0006.

As observed from the longitudinal data, widespread geographic differences in mean waitlist time and overall rate of bridging LRT use were observed between UNOS organ allocation regions. Analysis of geographical distribution of overall mean waitlist time identified UNOS Regions 1, 2, 4, 5, 8, and 9 as having high overall mean waitlist times (mean waitlist time >180 days), and UNOS Regions 3, 6, 7, 10, and 11 as having low overall mean waitlist times (Fig. 6A). Similar analysis of geographic distribution for bridging LRT rates identified UNOS Regions 2, 3, 4, 8,10, and 11 as high mean LRT regions (defined as >25% of patients with ≥1 LRT), compared to low mean LRT rates in Regions 1, 5, 6, 7, and 9 (Fig. 6B). Stratification of the study population by region and by both factors demonstrated significant differences in mean overall survival: 124.9 months in HWT, High LRT regions; 114.6 months in LWT, High LRT regions; 116.0 in HWT, Low LRT regions; and 114.8 months in LWT, Low LRT regions, *P* = 0.0006 (Fig. 7).

On multivariate Cox proportional hazards analysis of variables related to posttransplant survival, recipient age ≤65 years (hazard ratio, HR 1.37, 95% CI: 1.23–1.58, *P* < 0.001), donor age ≤45 years (HR 1.42, 95% CI: 1.30–1.66, *P* < 0.001), bridging LRT (HR 2.28, 95% CI: 1.39–3.14, *P* = 0.003) and high wait times (HR 2.37, 95% CI: 1.25–3.33, *P* < 0.001) were found to be significant independent predictors of post‐LT survival (Table 3).

**Table 3:** Multivariate logistic regression analysis of variables related to posttransplant survival

Prognostic factor	Parameters compared	Overall survival (OS) from orthotopic liver transplantation			
		Univariate analysis		Multivariate analysis	
		HR (95% CI)	*P*‐value	HR (95% CI)	*P*‐value
Age	>65 versus ≤65 years	1.71 (1.44–2.94)	**<0.001**	1.37 (1.23–1.58)	**<0.001**
Donor age	>45 versus ≤45 years	1.86 (1.67–2.19)	**<0.001**	1.42 (1.30–1.66)	**<0.001**
Gender	Male versus Female	0.80 (0.26–2.45)	0.61	N/A	–
Donor gender	Male versus Female	1.28 (0.41–3.98)	0.69	N/A	–
Body mass index (BMI)	>25 versus ≤25	1.02 (0.39–2.65)	0.95	N/A	–
Donor BMI	>25 versus ≤25	1.34 (0.48–3.43)	0.87	N/A	–
Bridging LRT	Present versus Absent	2.45 (1.93–2.49)	**<0.001**	2.28 (1.39–3.14)	**0.003**
Wait time	≤180 versus >180 days	2.69 (1.77–3.40)	**<0.001**	2.37 (1.25–3.33)	**<0.001**
Etiology of HCC	Hepatitis B/C versus OTHER	1.45 (0.33–6.44)	0.62	N/A	–
Warm ischemic time	>30 versus ≤30 min	1.80 (0.69–4.71)	0.23	N/A	–
Functional status at transplant	<70% versus ≥70%	3.12 (0.73–4.46)	0.08	N/A	–

Bold text indicates statistically significant *P*‐values

## Discussion

This study utilized a national transplantation registry to determine independent predictors of postliver transplant survival in MELD‐prioritized HCC recipients. In particular, we found that both pretransplant locoregional therapy and longer waitlist times (>180 days) independently predicted prolonged post‐LT survival for MELD‐prioritized HCC recipients. The association between higher waitlist times for HCC and prolonged post‐OLT survival found in the present analysis (mean survival, high vs. low wait times, 123.3 months vs. 120.4 months, *P* < 0.001) is in line with data from previous studies. A recent cohort study using the UNOS STAR registry examining 10,653 HCC and non‐HCC liver transplant candidates found that longer waiting times for MELD‐prioritized HCC patients predicted longer post‐OLT survival 19. It was hypothesized that longer wait times potentially selected for patients with more favorable tumor characteristics and that imposing a delay for listing and transplant in HCC patients may improve access and outcomes to LT 20, 21.

Our study found that LRT conferred a mean survival of 122.1 months in those who received pretransplant bridging LRT, as compared to 108.7 months in those who did not, *P* < 0.001. However, the currently available evidence for whether pretransplant LRT confers survival benefit in HCC patients remains heterogeneous and at times contradictory. Several studies assessing the benefits of pretransplant locoregional therapy have found that although locoregional therapy for HCC patients on the transplant list did not impact survival 22, 23, it may be useful to downstage selected patients and allow liver transplantation in patients who were otherwise ineligible 24. When pretransplant TACE in particular was investigated in a case–control study of 200 HCC patients by Decaens et al. 25, no significant differences between 5‐year survival rates with TACE (59.4%) and without TACE (59.3%) were observed. Similarly, a study on pretransplant RFA by DuBay et al. 26 showed no differences in 5‐year overall or tumor‐free survivals from list date or transplant, although analysis indicated that RFA allowed for maintenance on the waitlist for a longer period without negative effects on dropoff or survival.

Other studies have demonstrated significant survival benefits with LRT prior to transplant. Freeman et al., assessed liver transplantation within the United States from 1997 to 2006. In the subset of transplanted HCC patients in the MELD era, those who received ablation therapy pretransplant had a statistically significant improvement in 3‐year survival (79%) compared to those who were LRT naïve (75%) 27. Furthermore, Bharat et al. (2006) examined 100 patients who underwent OLT between 1985 to 2005 and found that in addition to significant downstaging in the LRT group at time of transplant (*P* = 0.008), the LRT group had significantly better 5‐year survival (82.4% vs. 51.8%; *P* = 0.01); it was observed that this improvement was limited to patients in patients with HCC stages II, III, and IV (77.6% versus 37.4%; *P* = 0.016). In comparison, this study data demonstrated a statistically significant survival benefit evident at 1‐year (LRT vs. non‐LRT, 95% vs. 94%), 3‐years (85% vs. 83%) and 5‐years post‐transplant (80% vs. 78%), *P* < 0.001 (*P* = 0.02). Further studies in the current literature suggest no statistical difference with bridging‐LRT 25, 28, 29, 30, 31, however, the majority of these studies were conducted at a single center, whereas the current observations of survival benefit with bridging LRT reflect large population data.

The existence of widespread geographic differences in waitlist time and bridging LRT rates observed in this study may be reflective of variations in practice patterns, organ availability and allocation policies between UNOS regions. However, such geographic differences may also suggest growing inequity in access to liver allocation and transplantation 32, 33. In examining waitlist outcomes, previous studies have indicated up to a 20‐fold disparity in transplant rates and as high as a 3.3‐fold increase in death rate between transplant programs and regions 34. Rana et al. 35 demonstrated that the mortality rate for candidates listed with a MELD score of 18 in Region 2 (31%) was more than double the mortality rate listed for candidates in Region 4 (13%). Furthermore, our study showed that stratification of the study population by region and by both LRT and wait time demonstrated significant differences in mean overall survival: 124.9 months in HWT, High LRT regions; 114.6 months in LWT, High LRT regions; 116.0 in HWT, Low LRT regions; and 114.8 months in LWT, Low LRT regions, *P* = 0.0006. Such significant differences in outcomes between regions further delineate the significant impact of longer wait times and locoregional therapies in prolonging post‐LT survival.

Our study includes several limitations. The retrospective nature of the study limits further delineation of the patient cohorts between groups. Although patient selection bias for LRT, such as functional status, social support or medical access, cannot be excluded, no significant differences in patient demographics and disease etiology were observed between the groups. These preliminary findings should be further investigated and validated in large randomized controlled studies.

In conclusion, we present an analysis of 14,511 MELD‐prioritized HCC patients who were listed for and received liver transplantation. Our study indicated that bridging LRT was a significant independent predictor for improved survival from OLT. Furthermore, our results confirm a survival advantage with longer waiting times in MELD‐prioritized HCC patients. This study offers promising results demonstrating that prolonged wait times and bridging locoregional therapy may benefit long‐term survival posttransplantation.

## Conflict of Interest

All authors have no financial or other disclosures relationship with any commercial organization that may have a direct or indirect interest in this manuscript.
